# Sorting by weighted inversions considering length and symmetry

**DOI:** 10.1186/1471-2105-16-S19-S3

**Published:** 2015-12-16

**Authors:** Christian Baudet, Ulisses Dias, Zanoni Dias

**Affiliations:** 1 Inria Erable Team, Université Claude Bernard Lyon I, Lyon, 69622, France; 2 Faculty of Technology, University of Campinas, Limeira, 13484-332, Brazil; 3 Institute of Computing, University of Campinas, Campinas, 13083-852, Brazil

**Keywords:** Genome Rearrangement, Inversion, Length, Symmetry

## Abstract

Large-scale mutational events that occur when stretches of DNA sequence move throughout genomes are called genome rearrangements. In bacteria, inversions are one of the most frequently observed rearrangements. In some bacterial families, inversions are biased in favor of symmetry as shown by recent research. In addition, several results suggest that short segment inversions are more frequent in the evolution of microbial genomes. Despite the fact that symmetry and length of the reversed segments seem very important, they have not been considered together in any problem in the genome rearrangement field. Here, we define the problem of sorting genomes (or permutations) using inversions whose costs are assigned based on their lengths and asymmetries. We consider two formulations of the same problem depending on whether we know the orientation of the genes. Several procedures are presented and we assess these procedure performances on a large set of more than 4.4 × 10^9 ^permutations. The ideas presented in this paper provide insights to solve the problem and set the stage for a proper theoretical analysis.

## Background

Among various large-scale rearrangement events that have been proposed to date, inversions were established as the main explanation for the genomic divergence in many organisms [1-3]. An inversion occurs when a chromosome breaks at two locations, and the DNA between those locations is reversed.

In some families of bacteria, an 'X'-pattern is observed when two circular chromosomes are aligned [2,4]. Inversions symmetric to the origin of replication (meaning that the breakpoints are equally distant from the origin of replication) have been proposed as the primary mechanism that explains the pattern [4]. The justification relies on the fact that one single highly asymmetric inversion affecting a large area of the genome could destroy the 'X'-pattern, although short inversions may still preserve it.

Darling, Miklós and Ragan [1] studied eight *Yersinia *genomes and added evidence that symmetric inversions are "over-represented" with respect to other types of inversions. They also found that inversions are shorter than expected under a neutral model. In many cases, short inversions affect only a single gene, as observed by Lefebvre *et al*. [5] and Sankoff *et al*. [6], which contrasts with the null hypothesis that the two endpoints of an inversion occur by random and independently.

Despite the importance of symmetry and length of the reversed segment, both have been somewhat overlooked in the genome rearrangement field. Indeed, the most important result regarding inversions is a polynomial time algorithm presented by Hannenhalli and Pevzner [3] that considers an unit cost for each inversion no matter its length or symmetry. When gene orientation is not taken into account, finding the minimum number of inversions that transform one genome into the other is a NP-Hard problem [7].

Some results have considered at least one of the concepts. There is a research line that considers the total sum of the inversion lengths as the objective function of a minimization problem. Several results have been presented both when gene orientation is considered [8,9] and when it is not [10-12].

Regarding symmetry, the first results were presented by Ohlebusch *et al *[13]. Their algorithm uses symmetric inversions in a restricted setting to compute an ancestral genome and, therefore, is not a generic algorithm to compute the rearrangement distance using only symmetric inversions. In 2012, Dias *et al*. presented an algorithm that considers only symmetric and almost-symmetric inversions [14]. They later included unitary inversions to the problem and provided a randomized heuristic to compute scenarios between two genomes that uses solely these operations [15].

Here we propose a new genome rearrangement problem that combines the concepts of symmetry and length of the reversed segments. Whereas previous works restricted the set of allowed operations by considering only inversions that satisfy constrains like symmetry or almost-symmetry [14,15], here we allow all possible inversions.

The problem we are proposing aims at finding low-cost scenarios between genomes when gene orientation is taken into account and when it is not, which is useful, among others, for building phylogenetic trees, annotating genomes or correcting already existing annotations. The results obtained are the first steps in exploring this interesting new problem.

## Definitions

Formally, a chromosome is represented as a *n*-tuple whose elements represent genes. If we assume no gene duplication, then this *n*-tuple is a permutation *π *= (*π*_1 _*π*_2 _≠ *π_n_*), 1 ≤ |*π_i_*| ≤ *n *and |*π_i_*| ↔ |*π_j_*| ↔ *i *≠ *j*. Because we focus on bacterial chromosomes, we assume permutations to be circular, and *π*_1 _is the first gene after the origin of replication.

We consider two cases depending on whether we know the orientation of the genes. If we know the orientation, then *π *is a signed permutation such that each element *π_i _*∈ {−*n*, −(*n *− 1), ..., −1, +1, +2, ..., +*n*}. If we do not know the orientation of the genes, then *π *is an unsigned permutation such that *π_i _*∈ {1, 2, ..., *n*}.

We treat permutations as functions such that *π*(*i*) = *π_i _*and *π*(−*i*) = −*π*(*i*). The inverse of a permutation *π *is denoted by *π*^−1^, for which $\pi_{\pi i}^{-1}=i$ for all 1 ≤ *i *≤ *n*. The composition between two permutations *π *and *σ *is similar to function composition in such way that *π*·*σ *= (*π*_*σ*(1) _*π*_*σ*(2) _⋯ *π*_*σ*(*n*)_).

Let *π *= (*π*_1 _*π*_2 _... *π_n_*) be a signed permutation, an inversion *ρ*(*i, j*), 1 ≤ *i *≤ *j *≤ *n *is an operation such that *π*·*ρ*(*i, j*) = (*π*_1 _⋯ *π*_*i*−1 _− *π*_*j *_− *π*_*j*−1 _⋯ − *π*_*i*+1 _⋯ − *π*_*i *_*π*_*j*+1 _⋯ *π*_*n*_). Let *π *= (*π*_1 _*π*_2 _⋯ *π_n_*) be an unsigned permutation, an inversion *ρ*(*i, j*), 1 ≤ *i *<*j *≤ n is an operation such that *π*·*ρ*(*i, j*) = (*π*_1 _⋯ *π*_*i*−1 _*π_j _π*_*j*−1 _⋯ *π*_*i*+1 _*π_i _π*_*j*+1 _⋯ *π_n_*).

Given two permutations *α *and *σ*, we are interested in finding rearrangement scenarios that link *α *to *σ*. Therefore, our scenarios are sequences of operations *ρ*_1_, *ρ*_2_, ..., *ρ_t _*such that *α*·*ρ*_1_·*ρ*_2_·... *ρ_t _*= *σ*.

Let *ι *= (1 2 ... *n*) be the identity permutation, sorting a permutation *π *= (*π*_1 _*π*_2 _... *π_n_*) is the process of transforming *π *into *ι*. Note that *σ*·*σ*^−1 ^= *σ*^−1^·*σ *= *ι_n_*. Thus, the scenario that transforms *α *into *σ *can be used to transform a permutation *π *into a permutation *ι *if we take *π *= *σ*^−1^·*α*. Therefore, we hereafter consider sorting permutations by inversions.

Previous researchers have worked on the inversion distance *d*(*π*) of an arbitrary permutation *π*, which is the minimum number of inversions that transform *π *into *ι*. Here we consider that each inversion *ρ*(*i, j*) has a cost which is based on the length and the symmetry of endpoints *i *and *j*.

The following functions help us to define our cost function and can be applied to identify any element *i *in the permutation *π*. **Position: ***p*(*π*, *i*) = *k *⇔ |*π_k_*| = *i*, *p*(*π*, *i*) ∈ {1, 2, ..., *n*}. **Sign: ***s*(*π*, *i*) = *k *⇔ *π_p_*(*π*, *i*) = *k *× *i*, *s*(*π*, *i*) ∈ {−1, +1}. **Slice: ***slice*(*π*, *i*) = min{*p*(*π*, *i*), *n *− *p*(*π*, *i*) + 1}, *slice*(*π*, *i*) ∈ {1, 2, ..., $\left\lceil \frac{n}{2}\right\rceil$}.

**Figure 1:**
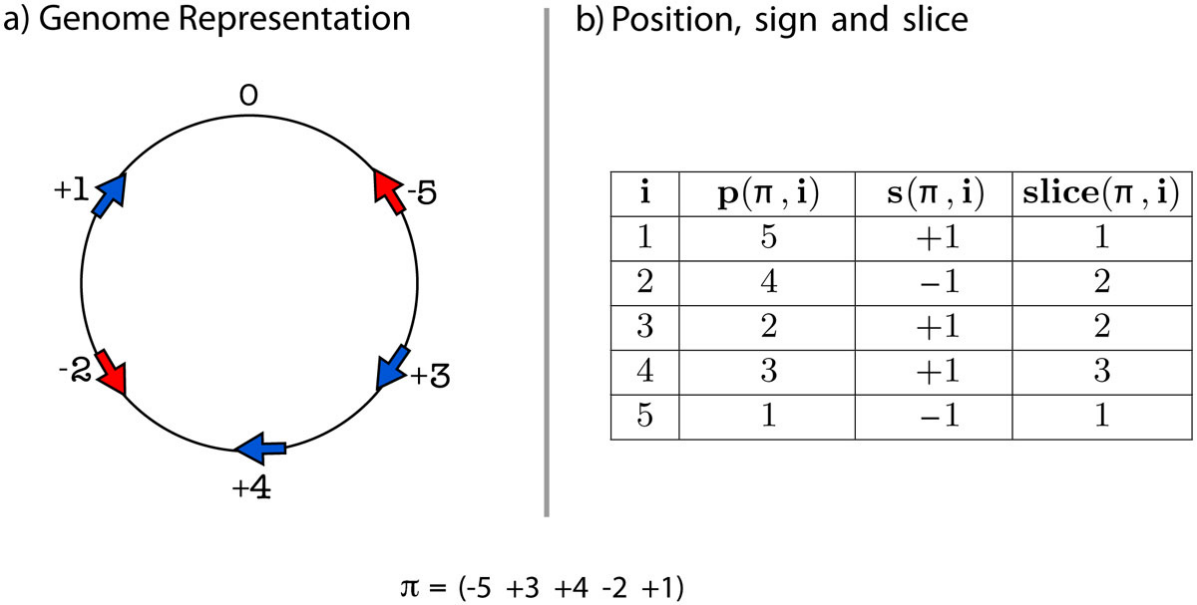
**(a) shows the genome representation for *π *= (−5 + 3 + 4 − 2 + 1) and (b) shows the values returned by three functions when applied to *π***.

Figure 1(b) shows the values of these functions for the signed permutation *π *= (−5 + 3 + 4 − 2 + 1). Note that the values for the functions ***slice ***and **position **would not change if instead we had the unsigned permutation *π *= (5 3 4 2 1).

**Figure 2:**
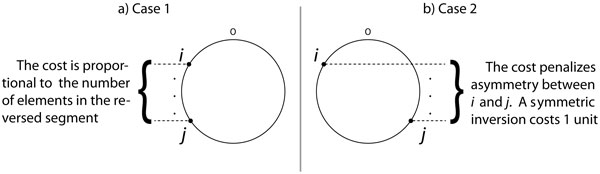
**Effect of the cost function when (a) **$i,\;j\leq \left\lceil \frac{n}{2}\right\rceil$**or **$i,\;j\geq \left\lceil \frac{n}{2}\right\rceil$**and (b) **$i>\left\lceil \frac{n}{2}\right\rceil$**and **$j<\left\lceil \frac{n}{2}\right\rceil$**, or **$j>\left\lceil \frac{n}{2}\right\rceil$**and **$i<\left\lceil \frac{n}{2}\right\rceil$.

Our cost function is: *cost*(*ρ*(*i, j*)) = |*slice*(*ι*, *i*) − *slice*(*ι*, *j*)| + 1. Its behavior is explained by looking at the two cases that arise. Figure 2 illustrates both cases.

**Case 1: **$i,j\leq \left\lceil \frac{n}{2}\right\rceil$**or **$i,j\geq \left\lceil \frac{n}{2}\right\rceil$.

In this case, the cost function can be simplified to cost(*ρ*(*i, j*)) = *abs*(*i*−*j*)+1, which means that it is proportional to the number of elements in the reversed segment. This cost is what one would expect from a length-weighted inversion distance in such a way that larger inversions cost more than short inversions.

**Case 2: **$i>\left\lceil \frac{n}{2}\right\rceil$**and **$j<\left\lceil \frac{n}{2}\right\rceil$, or $j>\left\lceil \frac{n}{2}\right\rceil$ and $i<\left\lceil \frac{n}{2}\right\rceil$.

In this case, the cost function is penalizing the asymmetry instead of the number of elements in the reversed segment. In effect, if the inversion *ρ*(*i, j*) is perfectly symmetric (meaning that *i *and *j *are equally distant from the origin of replication, so *slice*(*ι*, *i*) = *slice*(*ι*, *j*)), then the cost is given by cost(*ρ*(*i, j*)) = 1.

Therefore, our problem is to find a sequence of operations *ρ*_1_, *ρ*_2_, ..., *ρ*_t _such that *π*·*ρ*_1_·*ρ*_2 _... *ρ_t _*= *ι *and $\sum_{k=1}^{t}cost\left(\rho_{k}\right)$ is minimum.

## Methods

This section presents several greedy algorithms that take advantage from the characteristics of the cost function. The first greedy approach was named LR and constructs a solution by placing one element each time in the final position. After we place an element, we guarantee that we will not move it again.

Three other greedy approaches have been established, named NB, SMP and NB+SMP. These approaches rely on greedy functions to estimate how good an inversion might be. For each greedy function *f*, the benefit of an inversion *ρ *is the difference in the value returned by *f *before and after *ρ *is applied divided by the cost of *ρ*. For instance, let *π *be an arbitrary permutation, the benefit of *ρ *is computed as $ben_{f}\left(\pi ,\rho \right)=\frac{f\left(\pi \right)-f\left(\pi \cdot \rho \right)}{cost\left(\rho \right)}$. Note that we expect *f *to assign smaller values to permutations closer to the identity.

Using the greedy function *f*, we construct a sequence of inversions that sort *π *by iteratively adding an inversion with the best benefit among all possible inversions. The greedy function *f *guarantees a (possibly not optimum) solution if we can always find an inversion *ρ *such that *f *(*π*·*ρ*) <*f *(*π*) for any *π *≠ *ι*. However, our greedy functions presented in the following sections do not always guarantee that. Therefore, we study each case and we developed ways to circumvent this issue.

### Left or right heuristic

We use the term LR to refer to this approach as an acronym for Left or Right. We first divide the elements in the permutation in two groups. The first group refers to the elements that are in slices classified as sorted and the second group comprises those elements that are in unsorted slices. A slice *s *is in the sorted group if *p*(*π*, *s*) = *s*, *p*(*π*, *n *− *s *+ 1) = *n *− *s *+ 1, and the slices {1, 2, ..., *s *− 1} are also in the sorted group. Otherwise, *s *is in the unsorted group.

First, the *Left or Right *heuristic selects the least slice in the unsorted group. Then, we determine the element that should be moved first to that slice: the left or the right. The left (right) side is composed of the elements which are in positions that have indices bigger (lower) than the middle position.

To make this choice, we compute the total cost to put a given element in its final place. For signed permutations, we also consider the cost to place the element with a positive sign. After computing a cost for placing the left and the right elements, we choose the side that has the minimum cost. In case of tie, we move the right element.

If the slice has only one element that does not belong to it, we find the element that should be in that slice and perform an inversion to place it in the final position. After placing the element, we might have to change its sign on the signed version of our problem.

### Slice-misplaced pairs heuristic

We use the term SMP to refer to this heuristic as an acronym for Slice-Misplaced Pairs.

**Definition 1 ***We say that a pair *{*π_i_*, *π_j_*}, 1 ≤ *i *<*j *≤ *n*, *is slice-misplaced in π if slice*(*π*, *π_i_*) >*slice*(*π*, *π_j_*) *and slice*(*ι*, *π_i_*) <*slice*(*ι*, *π_j_*). *We use π_i _*~ *π_j _to represent that π_i _and π_j _are slice-misplaced*.

Let *SMP *(*π*) be the number of slice-misplaced pairs in *π *and Δ*_SMP _*(*π*, *ρ*) = *SMP *(*π*·*ρ*)−*SMP *(*π*) be the variation in the number of slice-misplaced pairs caused by an inversion *ρ*, then the benefit of an inversion is given by $ben_{SMP}=-\frac{\Delta_{SMP}\left(\pi ,\rho \right)}{cost\left(\rho \right)}$.

For some permutations *π *≠ *ι*, there is no inversion *ρ *such that *SMP *(*π*·*ρ*) <*SMP *(*π*). The following lemmas give us a better understanding from the properties of these permutations. We start by stating in Lemma 1 when we can be sure that at least one slice-misplaced pair can be removed.

**Lemma 1 ***Let π_i_, π_j _be two elements in π such that *|*i *− *j*| = 1 *and π_i _*~ *π_j_. Then there is at least one inversion ρ such that *Δ*_SMP _*(*π*, *ρ*) < 0.

*Proof *Let us assume, without loss of generality, that *slice*(*π*, *π_i_*) <*slice*(*π*, *π_j_*). Therefore, *slice*(*ι*, *π_i_*) >*slice*(*ι*, *π_j_*) since *π_i _*~ *π_j_*. The inversion *ρ *= *ρ*(*i, j*) if *i *<*j *or *ρ *= *ρ*(*j*, *i*) if *i *>*j *will remove the slice-misplaced pair {*π_i_*, *π_j_*} when creating the permutation *σ *= *π*·*ρ*. Let *π_k _*and *π*_l _be the elements in the same slice as *π_i _*and *π_j _*in *π*, respectively, the following statements suffice to conclude that Δ*_SMP _*(*π*, *ρ*) < 0. Note that it may occur that *π_j _*is the only element in the slice, therefore it suffices to prove the first statement.

• If *π_j _*≁ *π_k _*in *π*, then *π_i _*≁ *π_k _*in *σ*. We know that *slice*(*ι*, *π_j_*) >*slice*(*ι*, *π_k_*) since in *π *we have *π_j _*≁ *π_k _*and *slice*(*π*, *π_j_*) >*slice*(*π*, *π_k_*). Therefore, *π_i _*≁ *π_k _*in *σ *because *slice*(*ι*, *π_k_*) <*slice*(*ι*, *π_j_*) <*slice*(*ι*, *π_i_*) and *slice*(*σ*, *π_k_*) <*slice*(*σ*, *π_i_*).

• If *π_i _*≁ *π_l _*in *π*, then *π_j _*≁ *π_l _*in *σ*. We know that *slice*(*ι*, *π_i_*) <*slice*(*ι*, *π_l_*) since in *π *we have *π_i _*≁ *π_l _*and *slice*(*π*, *π_i_*) <*slice*(*π*, *π_l_*). Therefore, *π_j _*≁ *π_l _*in *σ *because *slice*(*ι*, *π_l_*) >*slice*(*ι*, *π_i_*) >*slice*(*ι*, *π_j_*) and *slice*(*σ*, *π_l_*) >*slice*(*σ*, *π_j_*).

Observe that we do not need to consider cases where *π_i _*~ π*_l _*or *π_j _*~ *π_k _*in *π*, because in the worst scenario these slice-misplaced pairs will not be removed.

**Lemma 2 ***Let π_i _be an element in π such that $slice\left(\iota ,\;\pi_{i}\right)=\left\lceil \frac{n}{2}\right\rceil$*. *If $slice\left(\pi ,\;\pi_{i}\right)\neq \left\lceil \frac{n}{2}\right\rceil$, then there is at least one inversion ρ such that *Δ*_SMP _*(*π*, *ρ*) < 0.   □

*Proof *If *π_i _*is the only element having $slice\left(\iota ,\;\pi_{i}\right)=\left\lceil \frac{n}{2}\right\rceil$ or if it occurs that $slice\left(\pi ,\;\pi_{i}\right)\geq slice\left(\pi ,\;\pi_{j}\right)$ for *π_i_*, *π_j _*such that $slice\left(\iota ,\;\pi_{i}\right)=slice\left(\iota ,\;\pi_{j}\right)=\left\lceil \frac{n}{2}\right\rceil$, then *π_i _*will form a slice-misplaced pair with all possible element *π_k _*such that *slice*(*π*, *π_k_*) >*slice*(*π*, *π_i_*). Therefore, it is straightforward from Lemma 1 that at least one inversion *ρ *such that Δ*_SMP _*(*π*, *ρ*) < 0 exists as long as $slice\left(\pi ,\;\pi_{i}\right)\neq \left\lceil \frac{n}{2}\right\rceil$.

If $\pi_{i}=\left\lceil \frac{n}{2}\right\rceil$ and |*i *- *j*| ≠ 1, it is straightforward from Lemma 1 that we can move *π_j _*toward the slice. When |*i *− *j*| = 1 we apply the inversion *ρ*(i, *k*) if *i *<*k *or *ρ*(*k*, *i*) if *k *<*i*, where *k *= *n *− *j *+ 1.   □

**Lemma 3 ***Let π *= (*π*_1 _*π*_2 _... *π_n_*) *be a permutation such that Lemmas 1 and 2 find no inversion that decreases the number of slice-misplaced pairs, then π has the form:*

$$For\;n\;odd\left\{\begin{array}{c} slice\left(i,\;\pi_{1}\right)\leq slice\left(i,\;\pi_{2}\right)\leq \ldots \leq \;slice\left(i,\;\pi_{\frac{n-1}{2}}\right)<slice\left(i,\;\pi_{\frac{n+1}{2}}\right)\\ slice\left(i,\;\pi_{\frac{n+1}{2}}\right)>slice\left(i,\;\pi_{\frac{n+1}{2}+1}\right)\geq \ldots \geq \;slice\left(i,\;\pi_{n-1}\right)\geq slice\left(i,\;\pi_{n}\right) \end{array}\right)$$

$$For\;n\;even\left\{\begin{array}{c} slice\left(i,\;\pi_{1}\right)\leq slice\left(i,\;\pi_{2}\right)\leq \ldots \leq \;slice\left(i,\;\pi_{\frac{n}{2}-1}\right)<slice\left(i,\;\pi_{\frac{n}{2}}\right)\\ slice\left(i,\;\pi_{\frac{n}{2}}\right)=slice\left(i,\;\pi_{\frac{n}{2}+1}\right)\\ slice\left(i,\;\pi_{\frac{n}{2}+1}\right)>slice\left(i,\;\pi_{\frac{n}{2}+2}\right)\geq \cdots \geq slice\left(i,\;\pi_{n-1}\right)\geq slice\left(i,\;\pi_{n}\right) \end{array}\right)$$

*Proof *Lemma 2 implies that if n is odd, then $\pi_{\frac{n+1}{2}}$ is in the highest slice in *ι*. The same occurs with the elements $\pi_{\frac{n}{2}}$ and $\pi_{\frac{n}{2}+1}$ that should be in the highest slice in *ι *if *n *is even. We know that *π_i _*≁ *π*_*i*+1_, otherwise one could find *ρ *such that Δ*_SMP _*(*π*, *ρ*) < 0 using the Lemma 1. That leads to the elements being ordered according to theirs slices in *ι *as shown.   □

**Lemma 4 **Δ*_SMP _*(*π*, *ρ*) = 0 *for any perfectly symmetric inversion ρ*(*i, j*) *such that j *= *n *− *i *+ 1.

*Proof *Inversions *ρ*(*i, j*) such that *j *= *n *− *i *+ 1 have no effect in the slice of any element in *π*. That said, no slice-misplaced pair will be created or removed whatsoever.

**Lemma 5 ***Let π be a permutation such that SMP *(*π*) = 0, *then slice*(*π*, *π_i_*) = *slice*(*ι*, *π_i_*) *for any *1 ≤ *i *≤ *n. In this case, perfectly symmetric inversions can sort π if it is unsigned and perfectly symmetric plus unitary inversions should be enough to sort π if it is signed*.

*Proof *Let *π_i _*be an element in *π *such that *slice*(*π*, *π_i_*) ≠ *slice*(*ι*, *π_i_*). There must exist an element *π_j _*such that *slice*(*π*, *π_j_*) = *slice*(*ι*, *π_i_*) ≠ *slice*(*ι*, *π_j_*). Two cases are possible: (*i*) *π_i _*~ *π_j_*, therefore *SMP *(*π*) > 0 or (ii) *π_i _*≁ *π_j_*, thus there must exist an element *π_k _*such that *slice*(*π*, *π_k_*) = *slice*(*ι*, *π_j_*) ≠ *slice*(*ι*, *π_k_*). In this second case, we can restart the entire process by calling *π_j _*and *π_k _*as *π_i _*and *π_j_*, respectively. Since we have a finite number of elements in the permutation, we know that the first case will be reached eventually.

If *slice*(*π*, *π_i_*) = *slice*(*ι*, *π_i_*) for any 1 ≤ *i *≤ *n*, we can reach the identity permutation by first performing perfectly symmetric inversions *ρ*(*i, j*), *slice*(*ι*, *i*) = *slice*(*ι*, *j*), if |*π_i_*| = *j *and |*π_j_*| = *i*. It requires at most $\left\lceil \frac{n}{2}\right\rceil$ inversions and each one will cost one unit. It should be enough for unsigned permutations, but for signed permutations we still need to perform unitary inversions *ρ*(*i*, *i*) if *s*(*π_i_*) = −1.

**Lemma 6 ***Let π *= (*π*_1 _*π*_2 _... *π_n_*) *be a permutation such that Lemmas 1 and 2 find no inversion that decreases the number of slice-misplaced pairs and SMP *(*π*) > 0, *then we can apply two inversions ρ*_1 _*and ρ*_2 _*such that SMP *(*π*) >*SMP *(*π*·*ρ*_1_·*ρ*2).

*Proof *Assuming no inversion as described by Lemmas 1 and 2 is possible, we know that *π *has the form of Lemma 3. Moreover, there is at least one pair of elements *π_i_*, *π*_*i *+1 _such that *slice*(*ι*, *π_i_*) = *slice*(*ι*, *π*_*i*+1_) and *slice*(*ι*, *π_i_*) ≠ $\left\lceil \frac{n}{2}\right\rceil$. We hereafter consider, without loss of generality, that (i) *slice*(*π*, *π_i_*) = *i *and *slice*(*π*, *π*_*i*+1_) = *i*+1; (ii) *slice*(*π*, *π*_*i*+1_) ≥ *slice*(*π*, *π_x_*) and *slice*(*π*, *π*_*i*+1_) ≥ *slice*(*π*, *π*_*x*+1_) for every pair *π_x_*, *π*_*x*+1 _such that *slice*(*ι*, *π_x_*) = *slice*(*ι*, *π*_*x*+1_); (iii) *π_j _*and *π*_*j*+1 _are elements that share the same *slice *with *π*_*i*+1 _and *π_i_*, respectively.

The first inversion we apply is *ρ*_1_(*i *+ 1, *j*). This inversion is perfectly symmetric and hence Δ*_SMP _*(*π*, *ρ*_1_) = 0 by Lemma 4. We assert that the inversion *ρ*_2 _applied after *ρ*_1 _will have Δ*_SMP _*(*π*·*ρ*_1_, *ρ*_2_) < 0 in order to prove the lemma. That said, we start by compiling attributes of *σ *= *π*·*ρ*_1 _and we will use these attributes to find *ρ*_2_.

• *a *= *slice*(*ι*, *π_i_*) = *slice*(*ι*, *σ_i_*)

• *a *= *slice*(*ι*, *π*_*i*+1 _) = *slice*(*ι*, *σ_j_*)

• *b *= *slice*(*ι*, *π_j_*) = *slice*(*ι*, *σ*_*i*+1_)

• *c *= *slice*(*ι*, *π*_*j*+1_) = *slice*(*ι*, *σ*_*j*+1_)

We know that *b *≥ *c *because *π *has the form described by Lemma 3, and we know that *a *≠ *b *and *a *≠ *c *because at most two elements can have a as slice in the identity permutation. Five different situations are possible:

1 *a *>*b *>*c*: we have *σ_i _*~ *σ*_*i*+1 _in *σ *because *a *>*b*. Therefore, the inversion *ρ*_2_(*i*, *i *+ 1) has Δ*_SMP _*(*π*·*ρ*_1_, *ρ*_2_) < 0 according to Lemma 1.

2 *b > c > a*: we have *σ_j _*~ *σ_j_*+1 in *σ *because *c > a*. Therefore, the inversion *ρ*_2 _(*i, i *+ 1) has ΔSMP (*π*·*ρ*_1_, *ρ*_2_) < 0 according to Lemma 1.

3 *b > a > c*: we show that this case is not possible. We have assumed that *slice*(*π*, *π*_*i*+1 _) ≥ *slice*(*π*, *π_x_*) and *slice*(*π*, *π*_*i*+1 _) ≥ *slice*(*π*, *π*_*x*+1_) for every pair *π_x_*, *π*_*x*+1 _such that *slice*(*ι*, *π_x_*) = *slice*(*ι*, *π*_*x*+1_). Therefore, the element *π*_*j*−1 _cannot have *slice*(*ι*, *π*_*j*−1_) = *b*, which forces us to conclude that there is one element *π_z _*such that *slice*(*ι*, *π_z_*) = *b *and *z *= *i *+ 2. However, since for each pair of elements *π_x_*, *π_y _*such that *slice*(*π*, *π_x_*) = *slice*(*π*, *π_y_*) >*b *cannot happen *π_x _*and *π_y _*on the same side, we must have more elements in one side of the permutation than in the other, which is impossible.

4 *b = c > a*: we have *σ_j _*~ *σ*_*j*+1 _in *σ *because *c > a*. Therefore, the inversion *ρ*_2_(*i, i *+ 1) has Δ*_SMP _*(*π*·*ρ*_1_, *ρ*_2_) < 0 according to Lemma 1.

5 *a > b = c*: we have *σ_i _*~ *σ*_*i*+1 _in *σ *because *a > b*. Therefore, the inversion *ρ*_2_(*i, i *+ 1) has Δ*_SMP _*(*π*·*ρ*_1_, *ρ*_2_) < 0 according to Lemma 1.

Lemmas 1 and 2 show cases such that at least one inversion will have positive benefit. No positive benefit inversion is guaranteed on permutations in the form described by Lemma 3, but in those cases we can use Lemmas 5 and 6 to assure that our greedy approach will eventually reach the identity permutation.

### Number of breakpoints heuristic

We use the term NB to refer to this heuristic as an acronym for Number of Break-points. Consider the extended permutation that can be obtained from *π *by inserting two new elements: *π*_0 _= 0 and *π*_*n*+1 _= *n *+ 1. The extended permutation is still denoted as *π*. Below, we present two definitions of breakpoint depending on whether we are dealing with signed or unsigned permutations.

**Definition 2 ***A pair of elements π_i_*, *π*_*i*+1 _*in a signed permutation π, with *0 ≤ *i *≤ *n, is a breakpoint if π*_*i*+1 _- *π_i _*≠ 1.

**Definition 3 ***A pair of elements π_i_*, *π*_*i*+1 _*in an unsigned permutation π, with *0 ≤ *i *≤ *n, is a breakpoint if *|*π*_*i*+1 _− *π_i_*| ≠ 1.

We use *NB*(*π*) to represent the number of breakpoints in a permutation and Δ*_NB _*(*π*, *ρ*) = NB(*π*·*ρ*) - *NB*(*π*) to represent the variation in the number of break-points caused by an inversion *ρ*.

The identity permutation *ι *is the only permutation with no breakpoints. Therefore, an inversion that decreases the number of breakpoints indirectly leads to the identity permutation. Since an inversion can affect only two breakpoints, we know that Δ*_NB _*(*π*, *ρ*) ∈ {−2, −1, 0, 1, 2}.

The benefit of an arbitrary inversion *ρ *can be computed as $ben_{NB}\left(\pi ,\;\rho \right)=-\frac{\Delta_{NB\left(\pi ,\rho \right)}}{cost\left(\rho \right)}$. We compute the benefit of all possible inversion and we choose one that maximizes the benefit.

Since breakpoint is a very common concept in the genome rearrangement field, previous publications have clearly stated which kind of permutations will not allow any inversion to decrease the number of breakpoints.

**Definition 4 ***Let π be an unsigned permutation, a strip of π is an interval *[*π_i_*, ... *π_j_*] *with no breakpoint such that *(*π*_*i*−1_, *π_i_*) *and *(*π_j _*, *π*_*j*+1_) *are breakpoints*.

Strips can be either increasing (*π_i _*<*π*_*i*+1 _< ... <*π_j_*) or decreasing (*π_i _*>*π*_*i*+1 _> ... >*π_j_*). Strips with only one element are considered decreasing strips. Kececioglu and Sankoff [16] proved that every unsigned permutation with a decreasing strip has at least one inversion that removes at least one breakpoint. Therefore, those inversions will have positive (non-zero) benefit. The same idea holds for signed permutations.

When we find a permutation *π *with no decreasing strips, we have no positive benefit. Therefore, we can use one of the following strategies to create decreasing strips as a contingency plan.

1. Use the Left or Right Heuristic known as LR.

2. The Left or Right Heuristic could break one strip because only a single element will be moved. Therefore, another approach is to compute the cost of placing the strip that contains the left element in the left side and the strip that contains the right element in the right side and hence use the less costly option.

3. Revert the entire unsorted group with one inversion.

4. Find the increasing strips and compute the cost of all possible inversions that reverse one or more of them. Note that we do not consider inversions that split any strip. After that, use the less costly inversion. We named this approach as the Best Strip strategy.

The Best Strip strategy leads to the best results in our experiments. Therefore, we use it in our comparative analysis.

### Number of breakpoints plus slice-misplaced pairs heuristic

We use the term NB+SMP to refer to this approach since it uses concepts retrieved from NB and SMP. We decided to favor breakpoints reduction in our greedy function: $\Delta_{NB+SMP}\left(\pi ,\;\rho \right)=\Delta_{NB}\left(\pi ,\;\rho \right)+\frac{\Delta_{SMP}\left(\pi ,\;\rho \right)}{n^{2}}$. We use the benefit computed as $ben_{NB+SMP}\left(\pi ,\;\rho \right)=-\frac{\Delta_{NB+SMP}\left(\pi ,\;\rho \right)}{cost\left(\rho \right)}$.

For unsigned permutations, we use the inversion *ρ *that maximizes the benefit if, and only if, $ben_{NB+SMP}\left(\pi ,\;\rho \right)>0$. Otherwise, we use the Best_Strip strategy in order to guarantee that the input permutation will be sorted.

For signed permutations, we compute the unsigned permutation *π*′ by removing the signs from *π *and we apply in *π *the inversion *ρ*′ that would be used in *π*′ according to the method previously described for unsigned permutations. In the end, if *π *≠ *ι*, we simply change the signs of negative elements with unitary inversions.

## Results and discussion

We implemented the algorithms using C++ Programming Language and experiments were executed at the cluster provided by the IN2P3 Computing Center (http://cc.in2p3.fr/).

Our source code is freely available at https://github.com/chrbaudet/SWI-LS.

We performed two batches of experiments. We first show experiments using small permutations and then we use considerably longer sequences up to size 100.

### Small permutations

We generated a dataset with all possible unsigned permutations up to size 12, which accounts for $\sum_{n=2}^{12}n!=522,956,312$ instances. We did the same for all possible signed permutations up to size 10, which accounts for $\sum_{n=2}^{10}2^{n}n!=3,912,703,160$ instances. Therefore, we have used a large dataset having more than 4.4 × 10^9 ^permutations.

For every permutation in the dataset, we were able to compute a minimum cost solution for comparison purposes. The minimum cost solution was calculated using a graph structure *G_n_*, for *n *∈ {1, 2, ..., 12}. We define *G_n _*as follows. A permutation *π *is a vertex in *G_n _*if, and only if, *π *has n elements. Let *π *and *σ *be two vertices in *G_n_*, we build an edge from *π *to *σ *if, and only if, there is an inversion *ρ *that transforms *π *into *σ*. The weight assigned to this edge is *cost*(*ρ*). Finally, we calculate the shortest path from *ι *to each vertex in *G_n _*using a variant of Dijkstra's algorithm for the single-source shortest-paths problem. This variant gives us the minimum cost to sort permutations in *G_n_*, as well as an optimum scenario of inversions.

Let *heu_cost *be the cost for sorting a permutation using some of our heuristics and *opt_cost *be the optimum cost, we can compute the approximation ratio as $\frac{heu\text{\_}cost}{opt\text{\_}cost}$.

**Figure 3:**
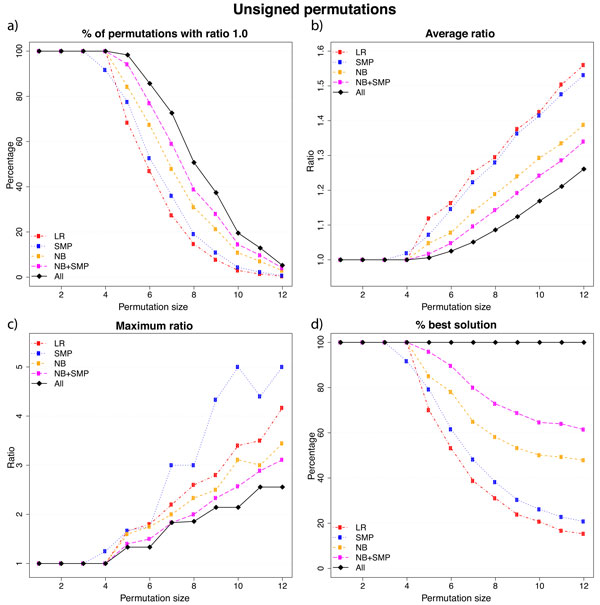
**Results for unsigned permutations**. In (a) we show how often each heuristic returns a minimum cost solution. In (b) and (c) we show the average and the maximum ratio, respectively. In (d) we show how often each heuristic succeeds in providing the best answer among all the heuristics.

**Figure 4:**
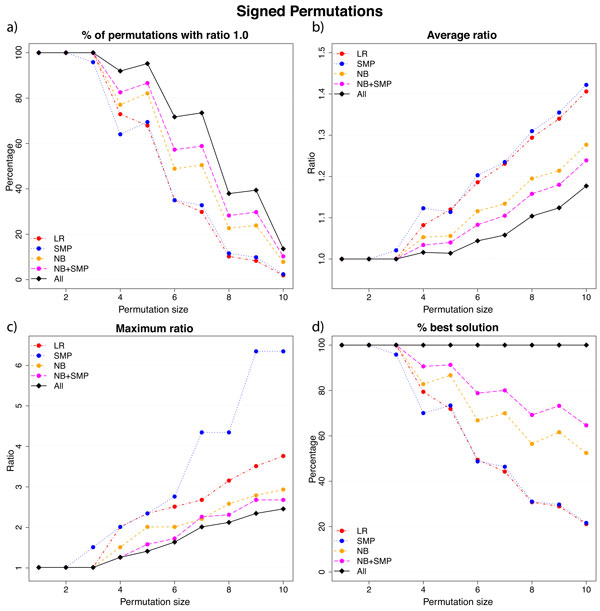
**Results for signed permutations**. In (a) we show how often each heuristic returns a minimum cost solution. In (b) and (c) we show the average and the maximum ratio, respectively. In (d) we show how often each heuristic succeeds in providing the best answer among all the heuristics.

Figures 3 and 4 summarizes our results for unsigned and singed permutations, respectively. The graphs in Figures 3(a) and 4(a) show how often each heuristic returns the optimum cost. The graphs in Figures 3(b) and 4(b) show the average ratio considering all permutations of a given size, while the graphs in Figures 3(c) and 4(c) exhibit the maximum ratios for the same group of permutations. These graphs are maximum or average values and they may not answer the question: for a single instance *π*, is there any algorithm that is likely to provide the best answer? The graphs in Figures 3(d) and 4(d) discuss this question by assessing the number of times each algorithm provides the least costly sequence. The values we present do not add up to 100% because of ties.

We observe that NB+SMP leads to the best results and it is consistently better than using just the number of breakpoints (NB) or just the variation in the number of slice-misplaced pairs (SMP) in every aspects we plot in Figures 3 and 4.

Individually, NB and SMP may be worse than NB+SMP, but NB returns results that are much closer to the optimum solution than SMP. That is true both on signed and unsigned permutations as we can see in Figures 3(b) and 4(b). The other graphs also corroborate this fact, which supports our decision of favoring the number of breakpoints when we compute Δ*_NB+SMP_*.

The simplistic approach LR leads to inferior results as reasonable. However, when we consider only the maximum ratio aspect, we observe that LR outdoes SMP. Indeed, the SMP approach is not consistent with respect to this aspect, which indicates that particular permutations are hard to sort using only the slice-misplaced pairs.

A final test checks if any profit is gained from running all possible heuristic. We added a new curve labeled as All, which selects for each instance the less costly result between those produced by our heuristics. As we can see, running all possible heuristics and keeping the best result accomplishes very good results. Indeed, it is consistently better than using solely the NB+SMP heuristic.

### Large permutations

We ran our algorithm on a set of arbitrarily large permutations. This set is composed of 190,000 random signed permutations and 190,000 random unsigned permutations. In both cases, the permutation size ranges from 10 to 1000 in intervals of 5, with 10,000 permutations of each size. Here, we do not have an exact solutions for these permutations. Therefore, we use the average cost instead of the approximation ratio to base our analysis.

**Figure 5:**
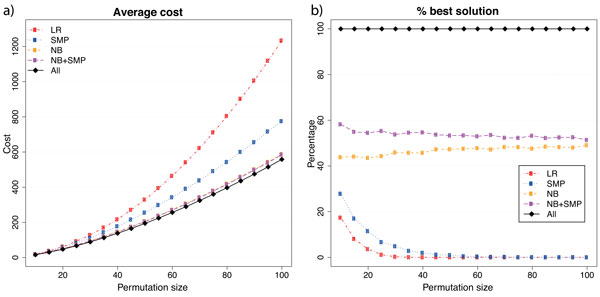
**Results for unsigned random permutations**. In (a) we show the average cost and in (b) we show how often each heuristic succeeds in providing the best answer among all the heuristics.

**Figure 6:**
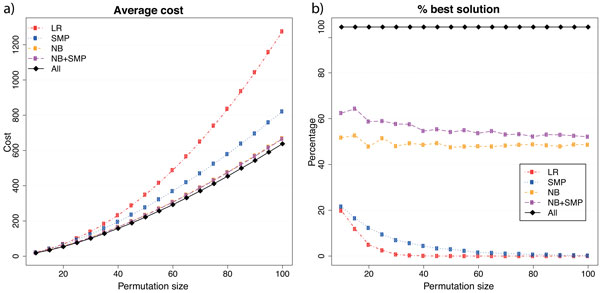
**Results for signed random permutations**. In (a) we show the average cost and in (b) we show how often each heuristic succeeds in providing the best answer among all the heuristics.

The analysis on random permutations reinforces the notion that NB+SMP leads to the best results. We observe in Figures 5 and 6 that the difference between NB and NB+SMP is very small on average. However, NB+SMP returns the less costly scenario in more cases.

Using random permutations allows us to draw information about the running time of each heuristic. In Table 1 we observe that LR is the fastest heuristic and the sorting scenario can be obtained almost instantly (less than 1 milliseconds), which is reasonable since this heuristic is very simplistic. The heuristic SMP and NB+SMP are the ones that take more time to finish, which can be explained, in part, by the fact that both need to compute slice-misplaced pairs. Since SMP returns scenarios with more inversions than NB+SMP, the former requires about twice the time used by the latter.

**Table 1:** Average running time for each permutation in milliseconds

	Unsigned Permutations				Signed Permutations			
**size**	**LR**	**SMP**	**NB**	**NB+SMP**	**LR**	**SMP**	**NB**	**NB+SMP**

10	0	0	0	0	0	0	0	0
20	0	3	0	2	0	4	0	2
30	0	24	2	15	0	34	3	17
40	0	110	9	63	0	149	10	69
50	0	356	22	188	0	468	23	204
60	0	938	50	466	0	1,210	51	497
70	0	2,110	88	994	0	2,681	89	1,048
80	0	4,259	148	1,922	0	5,464	150	2,068
90	0	7,980	231	3,457	0	9,959	230	3,624
100	0	13,876	349	5,843	0	17,155	345	6,106

## Conclusions

We have defined a new genome rearrangement problem based on the concepts of symmetry and length of the reversed segments in order to assign a cost for each inversion. The problem we are proposing aims at finding low-cost scenarios between genomes. We considered the cases when gene orientations is taken into account and when it is not. We have provided the first steps in exploring this problem.

We presented several heuristics and we assessed their performances on a large set of more than 4.4 × 10^9 ^permutations. The ideas we used to develop these heuristics together with the experimental results set the stage for a proper theoretical analysis.

As in other problems in the genome rearrangement field, we would like to know the complexity of determining the distance between any two genomes using the operations we defined. That seems to be a difficult problem that we intend to keep studying. We plan to design approximation algorithms and more effective heuristics.

## Competing interests

The authors declare that they have no competing interests.

## Authors' contributions

ZD came up with the idea of using the cost function that uses both symmetry and length of the affected segment. CB, ZD and UD designed the algorithms and the batches of experiments. UD and CB programmed an early prototype in python. After a series of iterative improvements, CB developed the final C++ code. UD drafted the manuscript. All authors read and approved the final manuscript.

## Declarations

This work was supported by CNPq grants (numbers 477692/2012-5 and 483370/2013-4), FAPESP grants (numbers 2012/01584-3, 2013/08293-7 and 2014/19401-8) and CAPES/COFECUB (number 831/15), by French Project ANR MIRI BLAN08-1335497 and the ERC Advanced Grant SISPHE (grant number [247073]10). The publication charges of this article were funded by FAPESP/CEPID grant 2013/08293-7.

This article has been published as part of *BMC Bioinformatics *Volume 16 Supplement 19, 2015: Brazilian Symposium on Bioinformatics 2014. The full contents of the supplement are available online at http://www.biomedcentral.com/bmcbioinformatics/supplements/16/S19
